# Prevalence and correlates of bullying and victimization among school students in rural Egypt

**DOI:** 10.1186/s42506-019-0019-4

**Published:** 2019-06-07

**Authors:** Yasmine Samir Galal, Maha Emadeldin, Maha Abdelrahman Mwafy

**Affiliations:** 1 Department of Public Health and Community Medicine, Faculty of Medicine, Cairo University, 31 Mohamed Hassan El-Gamal Street, 6th Zone, Nasr City, Cairo 11759 Egypt; 2 Department of Psychiatry, Faculty of Medicine, Beni-Suef University, 9/5, 216 Degla Street, Maadi, Cairo 111342 Egypt; 3 Department of Family Medicine, Faculty of Medicine, Cairo University, 94 American University Housing Fifth Settlement, Orman, Giza 651 Egypt

**Keywords:** Bullying, Victimization, Bully-victims, Risk factors, Behavioral disorders, Physical abuse

## Abstract

**Objectives:**

Knowledge on risk factors of bullying and victimization among school students is crucial for the implementation of preventive measures. This study was conducted to determine the prevalence and correlates of school bullying and victimization and their association with behavioral disorders among preparatory and secondary school students in rural Egypt.

**Study design:**

Cross-sectional

**Methods:**

A total of 476 students from two mixed public schools in rural Egypt (one preparatory and one secondary) were enrolled. A pretested self-administered questionnaire was used to collect sociodemographic characteristics and correlates of bullying and victimization including personal and social, family, school, and community factors. Frequency of bullying and victimization was measured using the short version aggression and victimization scale. The Strengths and Difficulties Questionnaire (SDQ) was used for screening behavioral problems.

**Results:**

Prevalence of bullying behavior was high (77.8%) among the studied group, of those 9.5% were unique bullies, 10.5% were unique victims, and 57.8% were bully-victims. On multivariate logistic regression analysis, failure in previous scholastic years (OR = 11.1, 95% CI 1.1–101.4, *P* = 0.033), witnessing family members using weapons (OR = 6.1, 95% CI 1.1–34.0, *P* = 0.038), male gender (OR = 2.3, 95% CI 1.1–5.0, *P* = 0.027), and mothers’ education (university or higher) (OR = 0.1, 95% CI 0.02–0.7, *P* = 0.017) remained the significant predictors for bullying. However, only having a drug addict friend (OR 2.5, 95% CI 1.1–5.4, *P* = 0.025) was the significant predictor for victimization. The independent predictors for being bully-victims in order of importance were exposure to physical violence in the street (OR = 5.1, 95% CI 1.2–22.7, *P* = 0.031), male gender (OR = 3.2, 95% CI 1.8–5.6, *P* < 0.001), witnessing fights (OR = 3.1, 95% CI 1.7–5.7, *P* < 0.001) and insulting words (OR = 2.5, 95% CI 1.3–4.7, *P* = 0.007) among family members, exposure to insulting words in the street (OR = 2.1, 95% CI 1.2–3.7, *P* = 0.010), watching violent movies (OR = 2.0, 95% CI 1.2–3.4, *P* = 0.008), and younger age (OR = 0.7, 95% CI 0.6–0.8, *P* < 0.001).

The self-reported SDQ revealed that the conduct problems scale scored significantly higher among bully-victims (2.8 ± 1.7 vs. 2.3 ± 1.6, *P* = 0.004).

**Conclusions:**

Prevalence of bullying behavior was high among rural adolescent school students. Establishment of a bullying prevention committee at school including all school personnel for addressing different factors associated with bullying behavior is recommended. Further follow-up and psychiatric assessment of students for predicting those prone to behavioral abnormalities are also recommended.

## Introduction

Violence in schools is a universal social problem which probably designates the most obvious form of juvenile violence manifested in the form of bullying and victimization which can exist regardless of the geographic location, socioeconomic status, and type of school [1]. Bullying is a subset of aggressive behavior, which occurs mainly between children and adolescents in schools. It involves repetitive and intentional use of power by one individual or group against another, causing physical or psychological damage [2]. Children can be directly involved in bullying either as bullies (i.e., perpetrators) or victims (i.e., targets) or bully/victims who are involved in bullying both as bullies and as victims [3]. Bullying behavior can take several forms including physical (i.e., fighting, pushing, hitting), verbal (i.e., teasing, calling names, threatening, spreading rumors), social (i.e., ignoring, exclusion, leaving on purpose), sexual (i.e., sexual comments, sexual harassment), and cyber (i.e., sending annoying electronic messages through the phone or computer) [4].

Involvement in school bullying poses a serious threat to the physical and psychological well-being of children [5]; victims are at increased risk of psychosomatic complaints such as headaches and abdominal pain, low self-esteem, and mental health disorders including anxiety, depression, loneliness, and suicide attempts, while bullies and bully-victims tend to have behavioral problems, delinquencies like alcohol and substance abuse, antisocial behavior, and criminal behavior later in life [6]. Furthermore, both victims and bullies are more likely to be rejected by their peers and to have poor academic achievement at school [7].

Although the prevalence of bullying varies markedly across countries, several studies indicated that it represents a common problem in elementary and secondary schools affecting up to half of children and adolescents worldwide [8]. The national study [9] conducted in 40 western countries reported that involvement in all 3 groups of bullying combined (i.e., bullying others, being bullied, and being both a bully and victim) ranged from 8.6 to 45.2% among boy and from 4.8 to 35.8% among girls. A study on the prevalence of school violence in Egypt reported 35% violent traits and 11.7% violent behavior among preparatory school students [10]. Another study conducted in Egypt found that 51% of boys and 20% of girls in preparatory and secondary schools had initiated violent attacks at schools [11].

There is no single cause associated with school violence; however, risk factors of bullying exist as a result of the interaction between the individuals and their environments including home, school, community, and society [12]. Knowledge on determinants and risk factors of bullying behavior is crucial for the identification of children at increased risk of becoming a bully or victim for proper implementation of prevention and control strategies [3].

In Egypt, most of the studies regarding school violence were mainly among children in urban communities [13, 14]. Furthermore, previous studies of school bullying in Egypt did not address the characteristics of adolescents who both bully and have been victimized (bully-victims). Hence, this study was conducted to determine the prevalence and correlates of all subgroups of school bullying (bullies, victims, bully-victims) and their association with behavioral disorders among preparatory and secondary school students in rural Egypt.

## Methods

### Participants

This cross-sectional study was conducted in Nikla rural village, Giza Governorate, Egypt. Preparatory and secondary school students aged 12 to 18 years from two mixed public schools (one preparatory and one secondary) were enrolled for the study while those known to have psychiatric disorders (previously or currently diagnosed with a psychiatric disease or on psychiatric medications) or not within this age group were excluded.

While orienting the local education directorate and taking permissions to conduct the study, a list of five districts was provided to conduct our survey (out of the 22 districts in Giza Governorate), out of them West Manshaat el Kannater was selected by simple random sampling. The district consists of 11 villages, of these Nikla village was selected by another simple random sampling method. It is populated with 25,717 (census of the year 2012). The village has four schools (three public and one private). Out of the three public schools, the two mixed schools (one preparatory and one secondary) were selected. Data were collected in the academic year 2016–2017 between April 2017 and June 2017 after taking the required administrative permissions from the school directors.

The sample size was calculated using the Epi Info software program version 7.1.5. (CDC Atlanta, GA, 2017). At 95% confidence interval and population size (number of students registered in the selected schools in the academic year 2016–2017 in preparatory and secondary grades) of 1090 students, assuming problem frequency of 30% [15] and 5% margin of error, the minimal sample size required was 251 participants. Using a design effect of 1.8 [16] to compensate for the error of the estimate encountered using cluster sampling instead of simple random sampling and adding 10% to compensate for potential non-response, the final total sample size was estimated to be 449 students. Out of 500 students approached, 476 agreed to participate (response rate of 95.2%). A sample of 476 students was selected from both schools by systematic random sampling (every 5th student was taken).

### Study instruments

1. A pretested interview questionnaire was used to collect the following data guided by the researchers:
  Sociodemographic characteristics including age, gender, parents’ education (illiterate, school, university, or higher) and working status (working/not working), birth order, number of siblings (two, three, or more), living with single or both parent(s), and crowding index (≥ 3, < 3).
  Risk factors for bullying/victimization: (a) personal and social factors including watching violent movies, carrying a weapon, having violent or drug addict friends, smoking and drug abuse, and exposure to sexual harassment; (b) family factors including witnessing fights, humiliating words, beating, threatening by/or using weapons among family members, and exposure to physical or verbal abuse by family members; (c) school factors including exposure to punishment at school (beating, mortification words, and others) and the association between school bullying and academic progress; and (d) community factors including exposure to physical violence, insulting words, or threatening by weapons in the street. Factors associated with bullying and victimization were derived from the available literature [17–19].
    Physical abuse of a child refers to the intentional use of physical force against the child, which is usually within the control of a parent or a person in a position of responsibility, power, or trust. It could result in harm for the child’s health, survival, or dignity. This includes hitting, beating, kicking, shaking, biting, strangling, burning, poisoning, and suffocating. There may be single or repeated incidents [20].
    Physical abuse at the family level usually refers to corporal punishment especially if it is from parents or other caregivers.
    Child sexual abuse is a form of child abuse in which an adult or older adolescent abuses a child for sexual stimulation. It refers to the involvement of a child in a sexual act aimed toward the physical gratification or the financial profit of the person committing the act [21].
    Verbal abuse of a child is also known as “verbal bullying.” It is the act of directing negative statements toward a child causing emotional harm. It consists of non-physical behaviors but which can still be damaging. It may take many forms; however, the most popularly used is name-calling. It also includes shouting, insulting, intimidating, threatening, and cursing [20].
2. The short version aggression and victimization scale [22] was used to detect the frequency of perpetration (i.e., bullying) or being a victim (i.e., victimization) of aggression during the week prior to the study. The scale has twelve questions, six for aggression and six for victimization, and responses can range from 0 times to 6 or more times per week referring to how many times specific behaviors occurred during the past 7 days [23]. Scale scores are additive; hence, each scale can range from 0 to 36 points. The aggression items assessed whether a child was the perpetrator of any of these six forms: teasing, pushing, name-calling, threatening, leaving another child on purpose, and making up stories. The victimization items assessed whether the child was victimized by other children by any of the formerly mentioned forms. The scale was translated from English to Arabic and back translated to English again by other independent experts, then followed by comprehensive revision by three professors of public health, psychiatry, and family medicine for its accuracy. Reliability testing was carried out; the internal consistency (Cronbach’s alpha) of the bullying scale was 0.77 and that of victimization was 0.75.
3. The self-reported version of Strengths and Difficulties Questionnaire (SDQ) is a brief screening questionnaire for behavioral problems including 25 questions comprising 5 scales of 5 items each: emotional symptoms, conduct problems, hyperactivity, peer relationship, and prosocial behavior. The overall internal consistency (Cronbach’s alpha) of all scales of the SDQ scale was 0.55. Each scale ranges from 0 to 10. The total difficulties score is generated by summing the scores from all the scales except the prosocial scale, creating a range from 0 to 40. The higher scores indicate more problems. The cut-off point of SDQ was regarded as 20 for adolescents [24].

### Procedures

Consent forms were sent to parents at home explaining the purpose and procedures of the study. After obtaining written informed consents from parents and adolescent assents from students, the researchers invited the children to a personal interview to complete the questionnaire under their guidance through a 30- to 45-min period during the recess. The students were told to read the instructions carefully, which informed them that honest answers were needed as that will be of great benefit for scientific research. Data collection process was conducted over 4 consecutive days by the three researchers where everyone was responsible for nearly 3–4 students/day through a period of 12 weeks to complete the selected sample. The data collection process was held in a quiet place with proper illumination and partitions between students to ensure privacy and without the presence of classroom teachers. The researchers clarified any misunderstanding in the questionnaire form and guided the students throughout the data collection process.

### Data analysis

Precoded data were entered on the computer using the Statistical Package for Social Science (SPSS) version 20. The data were summarized using mean and standard deviation for quantitative variables, while frequency and percentages were used for qualitative variables. Statistical differences between groups were tested using the chi-square test for qualitative variables and the independent sample *t* test for quantitative ones. Logistic regression analysis was done to identify the significant predictors of bullying behavior among students. Significance was considered at a *P* value of less than 0.05.

## Results

**Fig. 1:**
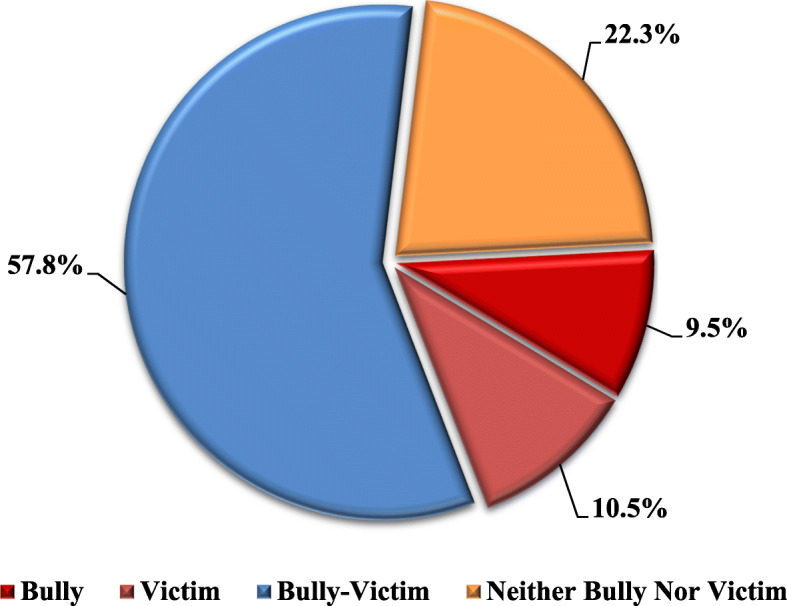
Pie chart showing the percentages of bullying subgroups among adolescent school students in rural Egypt

A total of 476 preparatory and secondary rural school students aged 12–18 years with a mean of 14.4 ± 1.8 years were enrolled for this study. The entire sample comprised 260 males (54.6%) and 216 females (45.4%). In this study, 370 students reported that they have been involved in some form of bullying in the past week. Hence, the prevalence of bullying was 77.8% among the studied group, of those 9.5% were unique bullies, 10.5% were unique victims, and 57.8% were bully-victims (Fig. 1).

Table 1 shows the association between self-reported bullying behavior and sociodemographic characteristics. The proportion of being a bully-victim was significantly associated with lower age (14.1 ± 1.6 vs. 14.9 ± 1.9, *P* < 0.001) and preparatory grade (64.2% vs. 50%, *P* = 0.001). The proportion of being a bully (10.8% vs. 7.9%, *P* = 0.004) or bully-victim (66.5% vs. 47.2%, *P* < 0.001) was significantly higher among males. There was a significant inverse association between mothers’ education and bullying behavior (*P* = 0.004); the prevalence of bullying was lower among students whose mothers had higher levels of education.

**Table 1:** Sociodemographic characteristics associated with bullying behavior among adolescent rural school students, Nikla rural village, Egypt

	Uninvolved (*n* = 106)	Bully (*n* = 45)	*P* value*	OR	95% CI	Victim (*n* = 50)	*P* value*	OR	95% CI	Bully-victim (*n* = 275)	*P* value*	OR	95% CI
Age	14.9 ± 1.9	14.9 ± 2	0.933^#^	1.0	0.8–1.2	14.3 ± 1.6	0.060^#^	0.8	0.7–1.02	14.1 ± 1.6	< 0.001^#^	0.8	0.8–0.9
Sex													
Male	39 (15.0)	28 (10.8)	0.004	2.8	1.4–5.8	20 (7.7)	0.700	1.1	0.6–2.3	173 (66.5)	< 0.001	2.9	1.8–4.6
Female	67 (31.0)	17 (7.9)	Ref	Ref	Ref	30 (13.9)	Ref	Ref	Ref	102 (47.2)	Ref	Ref	Ref
Birth order													
First	37 (20.8)	19 (10.7)	Ref	Ref	Ref	15 (8.4)	Ref	Ref	Ref	107 (60.1)	Ref	Ref	Ref
Middle	37 (21.5)	13 (7.6)	0.404	0.7	0.3–1.6	22 (12.8)	0.421	1.5	0.7–3.3	100 (58.1)	0.892	0.9	0.5–1.6
Last	23 (26.1)	7 (8.0)	0.337	0.6	0.2–1.6	8 (9.1)	0.806	0.9	0.3–2.3	50 (56.8)	0.422	0.8	0.4–1.4
Others	9 (23.7)	6 (15.8)	0.763	1.3	0.4–4.2	5 (13.2)	0.745	1.4	0.4–4.8	18 (47.4)	0.479	0.7	0.3–1.7
Number of siblings													
One or two	13 (15.7)	8 (9.6)	0.370	1.5	0.6–4.0	7 (8.4)	0.762	1.2	0.4–3.1	55 (66.3)	0.077	1.8	0.9–3.4
Three or more	93 (23.7)	37 (9.4)	Ref	Ref	Ref	43 (10.9)	Ref	Ref	Ref	220 (56.0)	Ref	Ref	Ref
Father education													
Illiterate or R and W	16 (19.8)	9 (11.1)	Ref	Ref	Ref	4 (4.9)	Ref	Ref	Ref	52 (64.2)	Ref	Ref	Ref
Prep or secondary	43 (22.5)	20 (10.5)	0.803	0.8	0.3–2.2	22 (11.5)	0.281	2.0	0.6–6.9	106 (55.5)	0.511	0.8	0.4–1.5
University or higher	47 (23.0)	16 (7.8)	0.432	0.6	0.2–1.6	24 (11.8)	0.284	2.0	0.6–6.8	117 (57.4)	0.517	0.8	0.4–1.5
Mother education													
Illiterate or R and W	37 (21.0)	23 (13.1)	Ref	Ref	Ref	20 (11.4)	Ref	Ref	Ref	96 (54.5)	Ref	Ref	Ref
Prep or secondary	46 (20.2)	20 (8.8)	0.355	0.7	0.3–1.5	25 (11.0)	1.000	1.0	0.5–2.1	137 (60.1)	0.607	1.1	0.7–1.9
University or higher	23 (31.9)	2 (2.8)	0.004	0.1	0.03–0.6	5 (6.9)	0.131	0.4	0.1–1.2	42 (58.3)	0.324	0.7	0.4–1.3
Grade													
Preparatory	45 (17.3)	19 (7.3)	0.979	1.0	0.5–2.0	29 (11.2)	0.070	1.9	0.9–3.7	167 (64.2)	0.001	2.1	1.3–3.3
Secondary	61 (28.2)	26 (12.0)	Ref	Ref	Ref	21 (9.7)	Ref	Ref	Ref	108 (50.0)	Ref	Ref	Ref
Father’s working status													
Working	102 (21.9)	45 (9.7)	0.187	NA	NA	49 (10.5)	0.557	1.9	0.2–17.7	269 (57.8)	0.474	1.6	0.5–6.4
Not working	4 (36.4)	0 (0)	Ref	Ref	Ref	1 (9.1)	Ref	Ref	Ref	6 (54.5)	Ref	Ref	Ref
Mother’s working status													
Working	6 (16.7)	2 (5.6)	0.760	0.8	0.2–4.0	4 (11.1)	0.727	1.4	0.4–5.4	24 (66.7)	0.319	1.6	0.6–4.0
House wife	100 (22.7)	43 (9.8)	Ref	Ref	Ref	46 (10.5)	Ref	Ref	Ref	251 (57.0)	Ref	Ref	Ref
Living with													
Both parents	96 (22.6)	43 (10.1)	0.511	2.2	0.5–10.7	47 (11.1)	0.552	1.6	0.4–6.2	239 (56.2)	0.326	0.7	0.3–1.4
Single parent	10 (19.6)	2 (3.9)	Ref	Ref	Ref	3 (5.9)	Ref	Ref	Ref	36 (70.6)	Ref	Ref	Ref
Crowding index													
≥ 3	35 (26.3)	14 (10.5)	0.819	0.9	0.4–1.9	14 (10.5)	0.583	0.8	0.4–1.7	70 (52.6)	0.139	0.7	0.4–1.1
< 3	71 (20.7)	31 (9.0)	Ref	Ref	Ref	36 (10.5)	Ref	Ref	Ref	205 (59.8)	Ref	Ref	Ref

*OR* univariate odds ratio, *CI* confidence interval, *Ref* reference category

*Chi-square test

^#^Independent sample *t* test

Table 2 depicts the association of bullying behavior with personal and family factors. Watching violent movies was significantly associated with being a bully (*P* = 0.028) or bully-victim (*P* < 0.001). There was a significant association between having a drug addict friend and all categories of bullying including bullies (*P* = 0.008), victims (*P* = 0.023), and bully-victims (*P* = 0.002). Moreover, the proportion of being a bully-victim was higher among drug-abusing students (64.2% vs. 55.2%, *P* = 0.002) and among those having a violent friend (67.4% vs. 49%, *P* < 0.001). Surprisingly, none of the three categories of bullying were significantly associated with smoking or sexual harassment.

**Table 2:** Personal and family factors associated with bullying behavior among the studied group

	Uninvolved (*n* = 106)	Bully (*n* = 45)	*P* value*	OR	95% CI	Victim (*n* = 50)	*P* value *	OR	95% CI	Bully-victim (*n* = 275)	*P* value *	OR	95% CI
Personal and social factors													
Watching violent movies													
Yes	50 (17.2)	30 (10.3)	0.028	2.2	1.1–4.6	25 (8.6)	0.741	1.1	0.6–2.2	186 (63.9)	< 0.001	2.3	1.5–3.7
No	56 (30.3)	15 (8.1)	Ref	Ref	Ref	25 (13.5)	Ref	Ref	Ref	89 (48.1)	Ref	Ref	Ref
Carrying a weapon													
Yes	4 (13.3)	3 (10.0)	0.426	1.8	0.4–8.5	2 (6.7)	0.945	1.1	0.2–6.0	21 (70.0)	0.172	2.1	0.7–6.3
No	102 (22.9)	42 (9.4)	Ref	Ref	Ref	48 (10.8)	Ref	Ref	Ref	254 (57.0)	Ref	Ref	Ref
Violent friend													
Yes	32 (14.1)	20 (8.8)	0.092	1.9	0.9–3.8	22 (9.7)	0.091	1.9	0.9–3.6	153 (67.4)	< 0.001	2.9	1.8–4.7
No	74 (29.7)	25 (10.0)	Ref	Ref	Ref	28 (11.2)	Ref	Ref	Ref	122 (49.0)	Ref	Ref	Ref
Smoking													
Yes	0 (0)	2 (18.2)	0.087	NA	NA	0 (0)	NA	NA	NA	9 (81.8)	0.067	NA	NA
No	106 (22.8)	43 (9.2)	Ref	Ref	Ref	50 (10.8)	Ref	Ref	Ref	266 (57.2)	Ref	Ref	Ref
Drug addict friend													
Yes	17 (12.4)	16 (11.7)	0.008	2.9	1.3–6.4	16 (11.7)	0.023	2.5	1.1–5.4	88 (64.2)	0.002	2.5	1.4–4.4
No	89 (26.3)	29 (8.6)	Ref	Ref	Ref	34 (10.0)	Ref	Ref	Ref	187 (55.2)	Ref	Ref	Ref
Drug abuse													
Yes	2 (7.4)	3 (11.1)	0.157	3.7	0.6–23.0	2 (7.4)	0.594	2.2	0.3–15.8	20 (74.1)	0.043	4.1	0.9–17.8
No	104 (23.2)	42 (9.4)	Ref	Ref	Ref	48 (10.7)	Ref	Ref	Ref	255 (56.8)	Ref	Ref	Ref
Sexual harassment													
Yes	11 (14.7)	7 (9.3)	0.369	1.6	0.6–4.4	7 (9.3)	0.593	1.4	0.5–3.9	50 (66.7)	0.063	1.9	0.96–3.8
No	95 (23.7)	38 (9.5)	Ref	Ref	Ref	43 (10.7)	Ref	Ref	Ref	225 (56.1)	Ref	Ref	Ref
Family factors													
Witnessing fights in family													
Yes	67 (19.6)	31 (9.1)	0.503	1.3	0.6–2.7	34 (9.9)	0.559	1.2	0.6–2.5	210 (61.4)	0.010	1.9	1.2–3.1
No	39 (29.1)	14 (10.4)	Ref	Ref	Ref	16 (11.9)	Ref	Ref	Ref	65 (48.5)	Ref	Ref	Ref
Insulting words													
Yes	17 (12.8)	11 (8.3)	0.224	1.7	0.7–4.0	15 (11.3)	0.044	2.2	1.01–5.0	90 (67.7)	0.001	2.5	1.4–4.5
No	89 (25.9)	34 (9.9)	Ref	Ref	Ref	35 (10.2)	Ref	Ref	Ref	185 (53.9)	Ref	Ref	Ref
Beating													
Yes	18 (16.1)	10 (8.9)	0.448	1.4	0.6–3.3	15 (13.4)	0.063	2.1	0.95–4.6	69 (61.6)	0.091	1.6	0.9–2.9
No	88 (24.2)	35 (9.6)	Ref	Ref	Ref	35 (9.6)	Ref	Ref	Ref	206 (56.6)	Ref	Ref	Ref
Threatening by weapons													
Yes	6 (10.7)	5 (8.9)	0.305	2.1	0.6–7.2	3 (5.4)	0.932	1.1	0.3–4.4	42 (75.0)	0.011	3.0	1.2–7.3
No	100 (23.8)	40 (9.5)	Ref	Ref	Ref	47 (11.2)	Ref	Ref	Ref	233 (55.5)	Ref	Ref	Ref
Using weapons													
Yes	2 (5.1)	5 (12.8)	0.025	6.5	1.2–34.9	2 (5.1)	0.594	2.2	0.3–15.8	30 (76.9)	0.004	6.4	1.5–27.1
No	104 (23.8)	40 (9.2)	Ref	Ref	Ref	48 (11.0)	Ref	Ref	Ref	245 (56.1)	Ref	Ref	Ref
Exposure to physical abuse													
Yes	14 (13.0)	7 (6.5)	0.703	1.2	0.5–3.2	12 (11.1)	0.091	2.1	0.9–4.9	75 (69.4)	0.004	2.5	1.3–4.6
No	92 (25.0)	38 (10.3)	Ref	Ref	Ref	38 (10.3)	Ref	Ref	Ref	200 (54.3)	Ref	Ref	Ref
Exposure to verbal abuse													
Yes	15 (12.2)	8 (6.5)	0.623	1.3	0.5–3.4	12 (9.8)	0.129	1.9	0.8–4.5	88 (71.5)	< 0.001	2.9	1.6–5.2
No	91 (25.8)	37 (10.5)	Ref	Ref	Ref	38 (10.8)	Ref	Ref	Ref	187 (53.0)	Ref	Ref	Ref

*OR* univariate odds ratio, *CI* confidence interval, *Ref* reference category

*Chi-square test

There was a significant association between bullying behavior and family factors in this study. The proportion of bully-victims was higher among students witnessing fights (61.4% vs. 48.5%, *P* = 0.010), humiliating words (67.7% vs. 53.9%, *P* = 0.001), and threatening by weapons between family members (75% vs. 55.5%, *P* = 0.011). Moreover, a significantly higher percent of bully-victims were exposed to physical abuse (69.4% vs. 54.3%, *P* = 0.004) and verbal abuse (71.5% vs. 53%, *P* < 0.001) by their family members. Witnessing family members using weapons during fights was significantly associated with being a bully (*P* = 0.025) or bully-victim (*P* = 0.004).

Regarding school factors and the academic performance of students, a significantly higher percent of bully-victims reported exposure to punishment at school (64.1% vs. 47.2%, *P* < 0.001). A significantly higher percent of bullies reported failure in previous scholastic years (27.8% vs. 8.7%, *P* = 0.009) and entering a second round (17.1% vs. 8.7%, *P* = 0.043) (Table 3).

**Table 3:** School and community factors associated with bullying behavior among the studied group

	Uninvolved (*n* = 106)	Bully (*n* = 45)	*P* value*	OR	95% CI	Victim (*n* = 50)	*P* value*	OR	95% CI	Bully-victim (*n* = 275)	*P* value*	OR	95% CI
School factors													
Punishment in school													
Yes	53 (17.8)	24 (8.1)	0.726	1.1	0.6–2.3	30 (10.1)	0.243	1.5	0.8–3.0	191 (64.1)	< 0.001	2.3	1.4–3.6
No	53 (29.8)	21 (11.8)	Ref	Ref	Ref	20 (11.2)	Ref	Ref	Ref	84 (47.2)	Ref	Ref	Ref
Tool of punishment													
Beating	24 (15.4)	17 (10.9)	0.077	3.8	0.9–15.0	13 (8.3)	0.797	0.9	0.3–2.4	102 (65.4)	0.344	1.5	0.7–3.0
Mortification words	22 (19.1)	7 (6.1)	0.719	1.7	0.4–7.8	16 (13.9)	0.801	1.2	0.4–3.2	70 (60.9)	0.849	1.1	0.5–2.3
Others°	16 (21.3)	3 (4.0)	Ref	Ref	Ref	10 (13.3)	Ref	Ref	Ref	46 (61.3)	Ref	Ref	Ref
Absence from school													
Yes	39 (28.3)	18 (13.0)	0.710	1.1	0.6–2.3	13 (9.4)	0.182	0.6	0.3–1.3	68 (49.3)	0.019	0.6	0.3–0.9
No	67 (19.8)	27 (8.0)	Ref	Ref	Ref	37 (10.9)	Ref	Ref	Ref	207 (61.2)	Ref	Ref	Ref
Failure in previous years													
Yes	1 (5.6)	5 (27.8)	0.009	13.1	1.5–115.9	1 (5.6)	0.540	2.1	0.1–35.0	11 (61.1)	0.126	4.4	0.6–34.3
No	105 (22.9)	40 (8.7)	Ref	Ref	Ref	49 (10.7)	Ref	Ref	Ref	264 (57.6)	Ref	Ref	Ref
Entered 2nd round													
Yes	5 (12.2)	7 (17.1)	0.043	3.7	1.1–12.4	3 (7.3)	0.712	1.3	0.3–5.6	26 (63.4)	0.130	2.1	0.8–5.6
No	101 (23.2)	38 (8.7)	Ref	Ref	Ref	47 (10.8)	Ref	Ref	Ref	249 (57.2)	Ref	Ref	Ref
Community factors													
Insulting words in street													
Yes	26 (14.4)	12 (6.6)	0.838	1.1	0.5–2.5	13 (7.2)	0.843	1.1	0.5–2.3	130 (71.8)	< 0.001	2.8	1.7–4.6
No	80 (27.1)	33 (11.2)	Ref	Ref	Ref	37 (12.5)	Ref	Ref	Ref	145 (49.2)	Ref	Ref	Ref
Physical violence in street													
Yes	2 (3.4)	4 (6.8)	0.065	5.0	0.9–28.8	4 (6.8)	0.084	4.5	0.8–25.6	49 (83.1)	< 0.001	11.3	2.7–47.2
No	104 (24.9)	41 (9.8)	Ref	Ref	Ref	46 (11.0)	Ref	Ref	Ref	226 (54.2)	Ref	Ref	Ref
Threatening by weapons													
Yes	1 (2.8)	4 (11.1)	0.028	10.2	1.1–94.4	3 (8.3)	0.097	6.7	0.7–66.1	28 (77.8)	0.002	11.9	1.6–88.6
No	105 (23.9)	41 (9.3)	Ref	Ref	Ref	47 (10.7)	Ref	Ref	Ref	247 (56.1)	Ref	Ref	Ref

*OR* univariate odds ratio, *CI* confidence interval, *Ref* reference category

*Chi-square test

^°^Teachers ignoring a student or not allowing him/her to attend their lessons

As regards community factors associated with bullying behavior, the prevalence of being a bully-victim was significantly higher among students exposed to insulting words (71.8% vs. 49.2%, *P* < 0.001), physical violence (83.1% vs. 54.2%, *P* < 0.001), and threatening by weapons (77.8% vs. 56.1%, *P* = 0.002) in the street (Table 3).

A backward stepwise logistic regression model was conducted to explore the predictors of the three categories of bullying behavior (Table 4). Variables entered in step 1 were the significant factors detected by univariate analysis. For bullying, the last step revealed that failure in previous scholastic years (OR = 11.1, 95% CI 1.1–101.4, *P* = 0.033), witnessing family members using weapons (OR = 6.1, 95% CI 1.1–34.0, *P* = 0.038), male gender (OR = 2.3, 95% CI 1.1–5.0, *P* = 0.027), and mothers’ education (university or higher) (OR = 0.1, 95% CI 0.02–0.7, *P* = 0.017) were the significant predictors. However, only having a drug addict friend (OR 2.5, 95% CI 1.1–5.4, *P* = 0.025) remained the significant predictor for victimization. The independent predictors for being bully-victims were found to be exposure to physical violence in the street (OR = 5.1, 95% CI 1.2–22.7, *P* = 0.031), male gender (OR = 3.2, 95% CI 1.8–5.6, *P* < 0.001), witnessing fights (OR = 3.1, 95% CI 1.7–5.7, *P* < 0.001) and humiliating words (OR = 2.5, 95% CI 1.3–4.7, *P* = 0.007) among family members, exposure to insulting words in the street (OR = 2.1, 95% CI 1.2–3.7, *P* = 0.010), watching violent movies (OR = 2.0, 95% CI 1.2–3.4, *P* = 0.008), and younger age (OR = 0.7, 95% CI 0.6–0.8, *P* < 0.001).

**Table 4:** Logistic regression model of factors associated with bullying behavior among the studied group

	Bully			Victim			Bully-victim		
	*P* value	OR	95% CI	*P* value	OR	95% CI	*P* value	OR	95% CI
Age							< 0.001	0.7	0.6–0.8
Gender (M/F)	0.027	2.3	1.1–5.0				< 0.001	3.2	1.8–5.6
Mother education (university or higher)	0.017	0.1	0.02–0.7						
Grade (Prep/secondary)							0.736	1.2	0.4–3.2
Watching violent movies	0.467	1.4	0.6–3.3				0.008	2.0	1.2–3.4
Violent friend							0.289	1.4	0.8–2.5
Drug addict friend	0.282	1.7	0.7–4.3	0.025	2.5	1.1–5.4	0.234	1.6	0.7–3.3
Drug abuse							0.282	2.6	0.5–14.9
Fights in family							< 0.001	3.1	1.7–5.7
Insulting words among family members				0.133	1.9	0.8–4.3	0.007	2.5	1.3–4.7
Threatening by weapons among family members							0.637	0.8	0.2–2.5
Using weapons by family members	0.038	6.1	1.1–34.0				0.153	3.7	0.6–22.2
Physical abuse by parents or other caregivers							0.605	1.2	0.6–2.6
Verbal abuse by parents or other caregivers							0.163	1.7	0.8–3.7
Punishment in school							0.981	1.0	0.6–1.8
Absence from school							0.141	0.6	0.3–1.2
Failure in previous years	0.033	11.1	1.2–101.4						
Entered 2nd round	0.252	2.4	0.5–10.3						
Insulting words in street							0.010	2.1	1.2–3.7
Physical violence in street							0.031	5.1	1.2–22.7
Threatening by a weapon in the street	0.345	3.3	0.3–37.9				0.245	3.6	0.4–31.8

*OR* multivariate odds ratio, *CI* confidence interval. Sensitivity of bullying model was 76.2%, and sensitivity of victimization model was 69.9%, while sensitivity of bully-victim model was 92%

The association between the three categories of bullying and behavioral problems among the study participants was presented in Table 5. No significant differences were observed between uninvolved students and those involved in bullying in all individual scales except the conduct problems scale which scored higher among bully-victims (2.8 ± 1.7 vs. 2.3 ± 1.6, *P* = 0.004).

**Table 5:** Association between bullying subgroups and behavioral disorders as per the Strengths and Difficulties Questionnaire among participants

	Uninvolved (*n* = 106)	Bully (*n* = 45)	*P* value^#^	OR	95% CI	Victim (*n* = 50)	*P* value^#^	OR	95% CI	Bully-victim (*n* = 275)	*P* value^#^	OR	95% CI
Prosocial scale	8 ± 1.8	8.1 ± 1.6	0.703	1.0	0.8–1.3	8 ± 1.9	0.927	1.0	0.8–1.2	7.9 ± 1.8	0.575	1.0	0.8–1.1
Hyperactivity scale	3.7 ± 1.9	3.5 ± 1.9	0.608	1.0	0.8–1.1	3.4 ± 1.9	0.417	0.9	0.8–1.1	4 ± 2	0.106	1.1	0.98–1.2
Emotional scale	4.7 ± 2.1	4.2 ± 2	0.167	0.9	0.7–1.1	4.7 ± 2.6	0.992	1.0	0.9–1.2	4.8 ± 2.1	0.862	1.0	0.9–1.1
Conduct problems scale	2.3 ± 1.6	2.4 ± 1.6	0.536	1.1	0.9–1.3	2.2 ± 1.8	0.824	1.0	0.8–1.2	2.8 ± 1.7	0.004	1.2	1.1–1.4
Peer problems scale	3.1 ± 1.6	3 ± 1.7	0.767	1.0	0.8–1.2	3.4 ± 1.9	0.344	1.1	0.9–1.4	3.2 ± 1.6	0.651	1.0	0.9–1.2
SDQ total scale	13.7 ± 5.4	13.2 ± 4.5	0.520	1.0	0.9–1.0	13.7 ± 5.8	0.979	1.0	0.9–1.1	14.8 ± 4.9	0.068	1.0	0.99–1.1

*OR* univariate odds ratio, *CI* confidence interval, *Ref* reference category

^#^Independent sample *t* test

## Discussion

School bullying and victimization have been recognized as major social and health problems worldwide, requiring the integrated efforts of the public, clinical practitioners, public health professionals, and educators [25]. Although it could be assumed that small communities and rural areas are protected from violence, there has been a dramatic rise in reported rural crime during the last few decades. The emergence of violence in rural schools is of particular concern in recent years [19]. Our findings revealed a markedly high prevalence of bullying behavior (77.8%) among adolescent rural school students. Among these, the highest prevalence was for bully-victims (57.8%) which could be explained by the greater likelihood of victims to turn into bullies in a way of expressing their anger. High rates of violence were also detected by another study conducted among elementary school children in Egypt, where the prevalence of physical violence was 69%, 82.8%, and 29% for victimization, witness of violence, and initiation of violent act, respectively [13]. However, a national survey conducted in 40 western countries (2009) [9] reported much lower rates of involvement in all the three groups of bullying combined (ranging from 4.8 to 45.2%). These variations in prevalence across countries could be attributed to methodological and cultural differences in defining the problem and to variations in target populations and instrumentation used.

Regarding sociodemographic factors associated with bullying behavior in the current study, the prevalence of being bully-victims was significantly associated with younger age and preparatory grade, which could reflect that older age was a protective factor for involvement in bullying. Similarly, other studies pointed out that bullying is more prevalent among students ranging from 11 to 13 years old, while prevalence from later childhood is reported comparatively rarely [26, 27]. Variation of prevalence according to age could be related to different physiological, biological, and psychological changes that accompany each stage of life. Male students in this study were more prone to be bullies and bully-victims, which could be attributed to cultural factors where boys in our community especially the rural are less often punished for misbehavior compared to girls. Similarly, the findings of Cook et al. [28] and Yang et al. [29] reported that bullying is more frequent in boys than girls. The fact that boys are more commonly involved in bullying does not necessarily mean they are more aggressive, but probably, they are more likely to adopt this behavior in an overt way (i.e.physical bullying), while girls are frequently involved in forms of bullying which may be difficult to identify like gossiping, teasing, rejecting, verbal threatening, and humiliating [30].

The finding that mothers’ education in the current study was inversely associated with bullying could be explained by the fact that the level of education plays an important role through its impact on the socioeconomic status on one hand and the behavior and lifestyle of children on the other. In agreement with our finding, Jansen et al. [3] reported that low educational level of parents was independently associated with the risk of children being bullies or bully-victims.

In this study, students watching violent movies and those having violent friends reported more involvement in bullying behavior as bullies and bully-victims, respectively. Similarly, Gentile et al. [31] reported that exposure to media violence is a risk factor for aggression and antisocial behavior. Moreover, Salmivalli et al. found that students who are engaged in a peer group involved in bullying show higher rates of bully perpetration [32]. In contrast, Larsen et al. stated that an aggressive youth is less likely to be susceptible to friends’ influence because he/she has already established a habit of aggression [33].

The relationship between drug abuse and bullying is well-documented in several studies [34, 35]. In the present study, students who reported being drug abusers were more prone to be bully-victims. A possible explanation is that substance abuse increases the risk of weapon carrying and being a victim or perpetrator of violence. Furthermore, having a drug addict friend was significantly associated with all groups of bullying. This is in consistence with the findings of a study conducted in Spain [36] which revealed that adolescent students who perceive that their friends have an easy access to drugs are more likely to be victims. Unexpectedly, exposure to sexual abuse in the current study was not significantly associated with bullying behavior. This could be explained by the conservative nature of the rural community where students are too shy or afraid to mention anything related to sexual abuse. In contrast, Duke et al. reported the association between childhood physical and sexual abuse and physical fighting together with other delinquencies [37].

Unfavorable familial conditions including living with a single parent, violence, and physical punishment among family members are significantly related to school bullying and victimization [26, 28]. The current study revealed that being a bully-victim was significantly more associated with experiencing fights and exposure to physical and verbal abuse at home, which may be explained by the stressful environment in which these children endure and also the possibility of imitating such aggressive behavior at school. This goes in line with another study conducted in Egypt [14], where a significant positive correlation was detected between verbal aggression among students and personal history of physical abuse.

Corporal punishment at schools markedly affects the school climate as victims may have the desire to displace their anger on teachers or other students and thus promoting aggressive behaviors [38]. In accordance, students who were exposed to punishment at school in the current study showed higher rates of being bully-victims. Similarly, Ez-Elarab et al. [13] found that corporal punishment was a risk factor of violence among public school students.

In the current study, the significant association between failure in previous scholastic years and bullying (*P* = 0.009) could be related to the fact that school failure causes suppression to students which reflects negatively on their behavior. Similarly, Nansel et al. [39] found that students involved in bullying and victimization are less academically engaged.

In this study, the significant association between being bully-victims and students’ exposure to physical violence in the street could be explained by the idea that exposure to hostile interactions in the neighborhood encourages students to imitate such behaviors especially among their peers [40]. In the same context, Cook et al. reported that characteristics of neighborhoods have a detrimental effect on bullying behavior, where living in a safe neighborhood predicted less bullying and victimization [28].

After conducting a multivariate logistic regression model in the current study, the most predicting factor for being a bully was failure in previous scholastic years (OR = 11.1, 95% CI 1.1–101.4, *P* = 0.033), followed by witnessing family members using weapons. Male students were 2.3 times at a higher risk of being a bully. However, mothers’ education (university or higher) was the least predicting variable for bullying. Students having a drug addict friend were 2.5 times more prone to be victims (OR = 2.5, 95% CI 1.1–5.4, *P* = 0.025). Furthermore, the most significant predictors in order of importance for being bully-victims were exposure to physical violence in the street (OR = 5.1, 95% CI 1.2–22.7, *P* = 0.031), male gender, and witnessing fights among family members; however, younger age was the least predicting variable (OR = 0.7, 95% CI 0.6–0.8, *P* < 0.001). These results revealed that in comparison with past studies of urban youth, nearly similar factors predict bullying behavior among urban and rural youth. In another study conducted in Egypt, risk factors of violence in schools detected by multivariate analysis were absence of attachment figure as a father, mother, and teacher; mode of delivery; living with a single parent; low school marks; and corporal punishment [13]. In a study conducted in Spain, risk factors of peer school victimization detected on multiple logistic regression analysis were being male, school adaptation (students rejected by their peers), social maladjustment, and perception of the friends’ attitude toward access to drugs (students who perceive that their friends would have a moderate or easy access to drugs) [36].

Bullying and victimization do not only affect the physical status of students but their emotional, psychological, and social well-being as well which consequently affect different aspects of their behavior [13]. The SDQ tool which was used in this study for probing the relation between bullying/victimization and behavioral problems among students revealed that only bully-victims scored significantly higher in the conduct problems scale. This could be explained by the idea that the tool used for detecting bullying behavior depended on measuring the frequency of initiation or exposure to violence in the past 7 days only which may not permit precise estimation of the problem. In the study conducted in Egypt by Ez-Elarab et al., SDQ revealed highest abnormal score in the total score and in the emotional, conduct, and hyperactivity problems as well in victimized students [13].

### Limitations of the study

First, the tool used for detecting bullying and victimization depends on the frequency of being a bully, a victim, or a bully-victim in the past 7 days only which may not be an accurate estimation of the problem; second, this study is cross-sectional which eliminates the causal relationship of the data; and third, behavioral problems were identified using the self-reported version of the SDQ which was a single informant assessment from the students and not from the teachers or the parents, which may not have provided a complete picture of the problem. Finally, no direct comparison with non-rural students was done; we relied only on findings from previous studies of urban youth to compare predictors across communities.

## Conclusion

Prevalence of bullying behavior was high among adolescent rural school students reflecting the importance of implementing effective intervention programs in rural schools which should focus on identifying students with risk factors including family, school, and community together with personal and social factors. Given the finding that punishment at school was found to be significantly associated with higher proportion of bully-victims, supervising teachers’ behavior and promoting better interaction between students and teachers are recommended. Establishment of a bullying prevention committee at school including all school personnel and involving parents as well is recommended. Students themselves should be encouraged to actively participate in the supervision and prevention of bullying. The high conduct problems scale among bully-victims necessitates further follow-up of students for predicting those at higher risk of behavioral abnormalities.

## Data Availability

The datasets used and/or analyzed during the current study are available from the corresponding author on reasonable request.

## Abbreviations

CI: Confidence interval
OR: Odds ratio
Ref: Reference category
SDQ: Strengths and Difficulties Questionnaire
SPSS: Statistical Package for Social Science
